# First successful treatment of *Clostridium perfringens*-associated emphysematous hepatitis: a case report

**DOI:** 10.3389/fmed.2023.1164466

**Published:** 2023-05-17

**Authors:** Christian Bayerl, Ann-Kathrin Berg, Stefan Angermair, Damon Kim, Bernd Hamm, Katharina Beyer, Christian Schineis

**Affiliations:** ^1^Charité – Universitätsmedizin Berlin, Corporate Member of Freie Universität Berlin and Humboldt-Universität zu Berlin, Department of Radiology, Berlin, Germany; ^2^Charité – Universitätsmedizin Berlin, Corporate Member of Freie Universität Berlin and Humboldt-Universität zu Berlin, Department of Surgery, Berlin, Germany; ^3^Charité – Universitätsmedizin Berlin, Corporate Member of Freie Universität Berlin and Humboldt-Universität zu Berlin, Department of Anesthesiology and Intensive Care Medicine, Berlin, Germany

**Keywords:** case report, emphysematous hepatitis, computed tomography, liver resection, septic shock, *Clostridium perfringens*

## Abstract

Emphysematous diseases of the abdomen are rare with an often inconspicuous presentation of symptoms and rapid lethal outcome if untreated. We report the first successfully treated case of *Clostridium perfringens*-associated emphysematous hepatitis. In the emergency room, a 79-year-old man presented with shortness of breath and deteriorated general condition since the morning of admission. Initial CT scans showed a small but rapidly expanding gas collection in liver segment 6. Emergency surgery with atypical liver resection was performed immediately. With early resection and prolonged administration of antibiotics in the presence of sepsis, the patient recovered successfully and was discharged 37 days after admission. As in our case, prompt diagnosis with early surgical treatment is crucial for the management of emphysematous hepatitis.

## Introduction

Emphysematous hepatitis is a very rare and rapidly progressive infectious condition characterized by hepatic gas accumulation without fluid components or mass effect and very high mortality (1–10). Only a few cases of this disease have been reported globally (1–14). To the best of our knowledge, this is the first successfully treated patient with *Clostridia*-associated emphysematous hepatitis who has survived.

## Case presentation

A 79-year-old male patient presented to our emergency department with shortness of breath. According to his wife, his general condition had deteriorated since the morning of admission.

He had a medical history of hypertension, hyperlipidemia, non-insulin-dependent diabetes mellitus (NIDDM), benign prostate hyperplasia, atrial fibrillation, and chronic heart failure with an ejection fraction of 30–35% as a result of myocardial infarctions 33 and 7 years before admission with percutaneous coronary intervention, implantation of two drug-eluting stents in the right coronary artery and implantation of a CRT-D. His daily medication included 25 mg of spironolactone, 5 mg of bisoprolol, 100 mg of acetylsalicylic acid, 20.7 mg of atorvastatin, 0.4 mg of tamsulosin, 12.3 mg of dapagliflozin and 3 mg of phenprocoumon.

On examination, the tympanic temperature was 38.5°C, the blood pressure was 137/117 mm Hg, and the pulse was 109 beats per minute. He had tachypnoea of 24 breaths per minute and oxygen saturation of 94 % on ambient air. In the initial physical examination, the abdomen was tender without abdominal guarding. Laboratory tests showed an elevated white blood count of 14,500 per cubic millimeter (reference range: 3,900 to 10,500), a CRP level of 39.7 milligrams per liter (reference: < 5.0), abnormal kidney function (creatinine 2.19 milligrams per decilitre, reference range: 0.70–1.20), and liver function with elevated liver enzymes (alanine aminotransferase of 70 U per liter, reference: < 41; aspartate aminotransferase of 64 U per liter, reference: < 50; alkaline phosphatase of 145 U per liter, reference range: 40–130; gamma-glutamyl transferase of 283 U per liter, reference range: 8–61) and bilirubin (total b. of 2.26 milligrams per decilitre, reference: < 1.20; conjugated b. of 1.25 milligrams per decilitre, reference: < 0.30). His blood glucose level was 225 milligrams per deciliter (reference range: 82–115).

**Figure 1:**
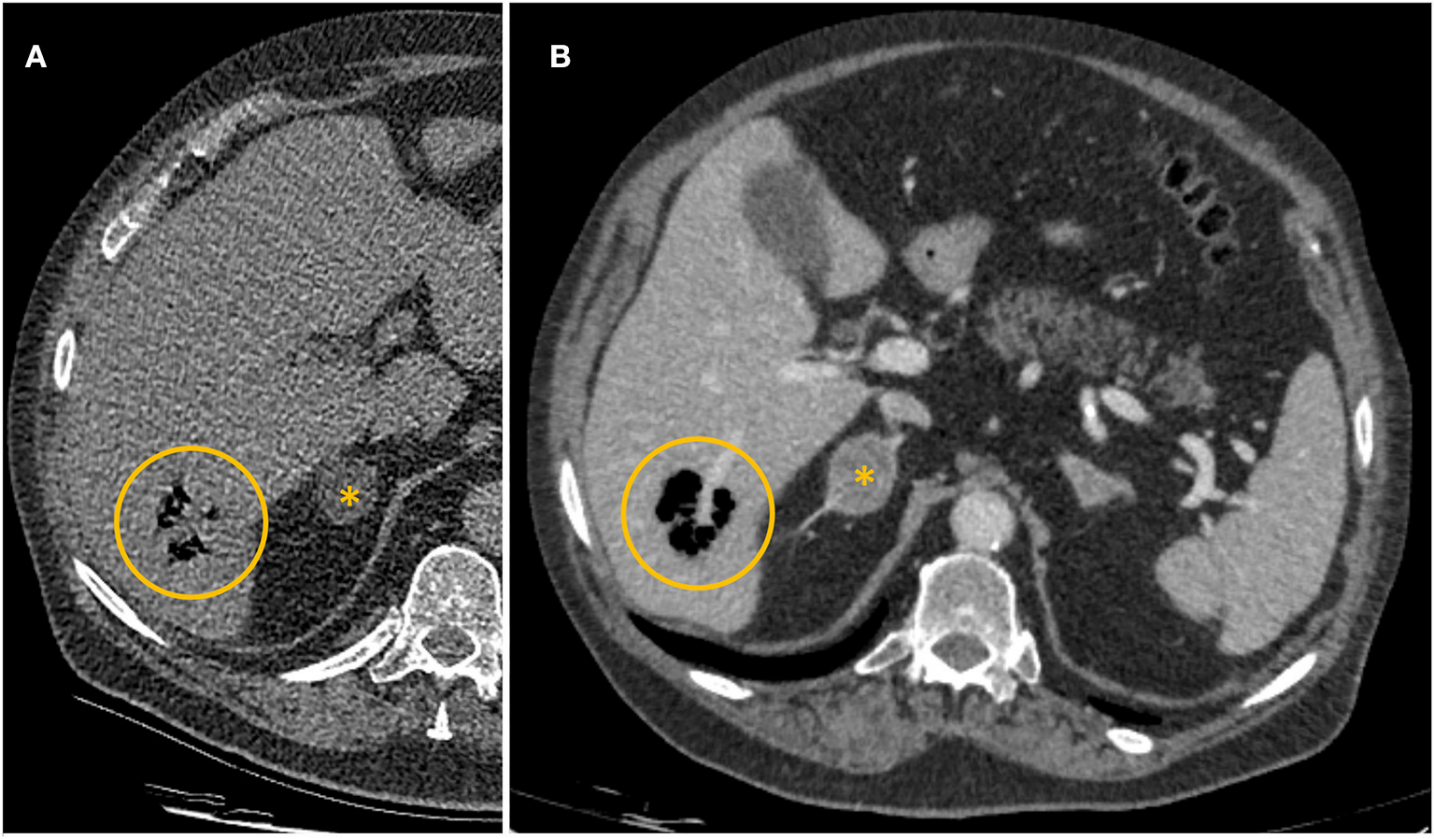
**(A)** Axial computed tomography pulmonary angiogram on admission. Collection of gas in liver segment 6 (yellow circle). **(B)** Contrast-enhanced computed tomography of the abdomen 154 min after the first CT scan. Collection of gas in liver segment 6 (yellow circle). Incidental finding of a mass of the right adrenal gland (^*^).

Due to increasing tachypnoea, tachycardia, and progressive hypotension, pulmonary artery embolism was the most likely diagnosis at that point. Therefore, a pulmonary CT angiogram was performed, which showed no evidence of pulmonary artery embolism or relevant abnormalities in the lungs. Purely by chance, the scan found a small, circumscribed gas collection in liver segment 6 with a diameter of 34 millimeters (Figure 1). The patient had no known history of liver or biliary tract disease.

After the scan, the patient's condition deteriorated rapidly, and he subsequently developed a distributive shock, requiring vasopressor support. After stabilization, a contrast-enhanced CT scan of the abdomen was performed 154 min after the initial thoracic scan. It showed marked enlargement of the periportal gas collection in liver segment 6 from 34 to 40 mm in diameter with well-described margins, no fluid collections, and no evidence of abscess formation (Figure 1). Volumetric analysis of the gas collection showed a 4 fold increase between the two scans. No relevant hepatic vascular or perfusion disorder, especially no gas in the portal vein and its branches could be detected in the acquired delayed phase. Nevertheless, intrahepatic and extrahepatic bile ducts were dilated due to a suspected stricture of the ampulla of Vater. This led to the diagnosis of emphysematous hepatitis with acute liver failure, most likely due to altered drainage of the common bile duct, resulting in changes in clinical presentation and laboratory studies.

Immediately after the second CT scan, the patient was admitted to the interdisciplinary intensive care unit of our hospital and prepared for exploration and non-anatomic liver resection.

**Figure 2:**
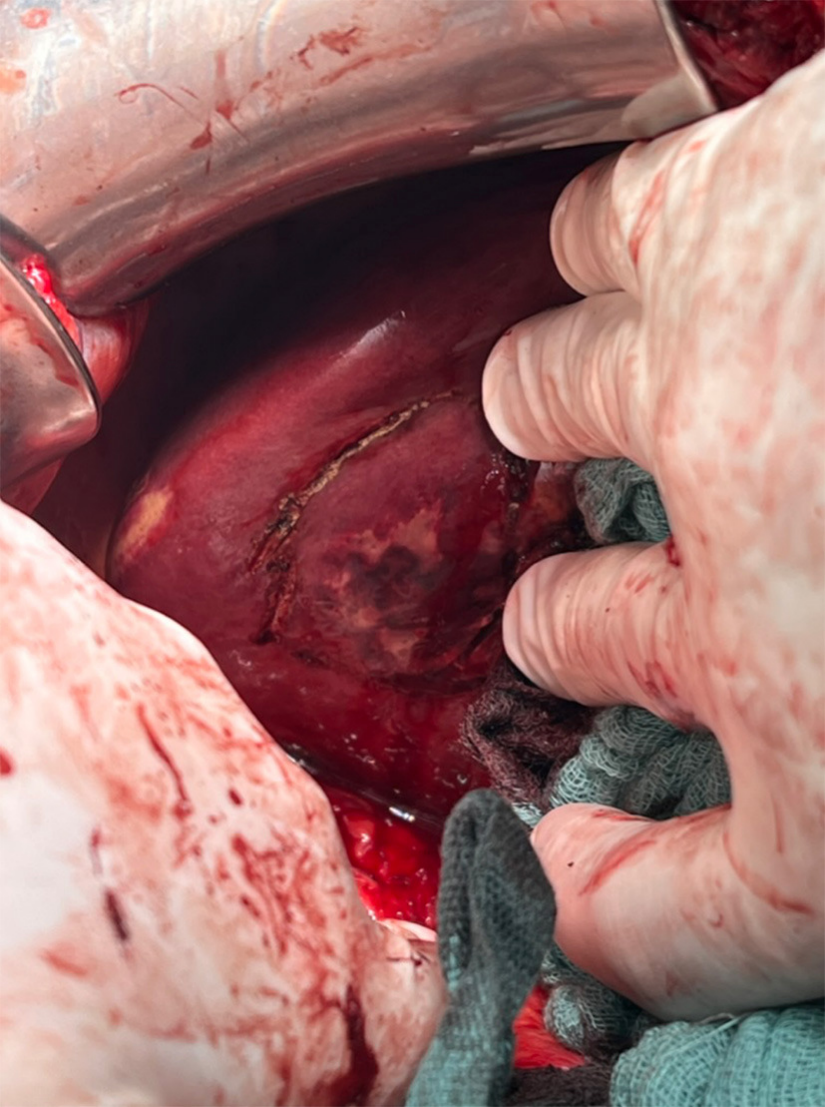
View from caudal to segment 6. Palpable affection marked with monopolar cautery.

Intraoperatively, access was obtained via a costal margin incision. First, cholecystectomy and partial mobilization of the right liver were performed to gain better exposure. The affected liver tissue presented deliquescent underneath an intact liver capsule (Figure 2). The capsule was incised and surgical debridement and atypical liver resection with adequate safety margins were performed by monopolar and bipolar dissection and situational ligation of prominent bile ducts or hepatic vessels. A swab was taken for microbiological testing. During liver debridement and atypical resection, a hemorrhage occurred from a distal branch of the portal vein that ran right through the affected tissue. A 5-min pringle maneuver was applied to control the bleeding by placing a suture around the portal branch. Due to intrahepatic and extrahepatic cholestasis, bile duct revision with insertion of a T-drain was performed to drain bile extra-corporally. Choledocholithiasis could not be detected intraoperatively. After the procedure, the patient was readmitted to the intensive care unit for further stabilization.

**Figure 3:**
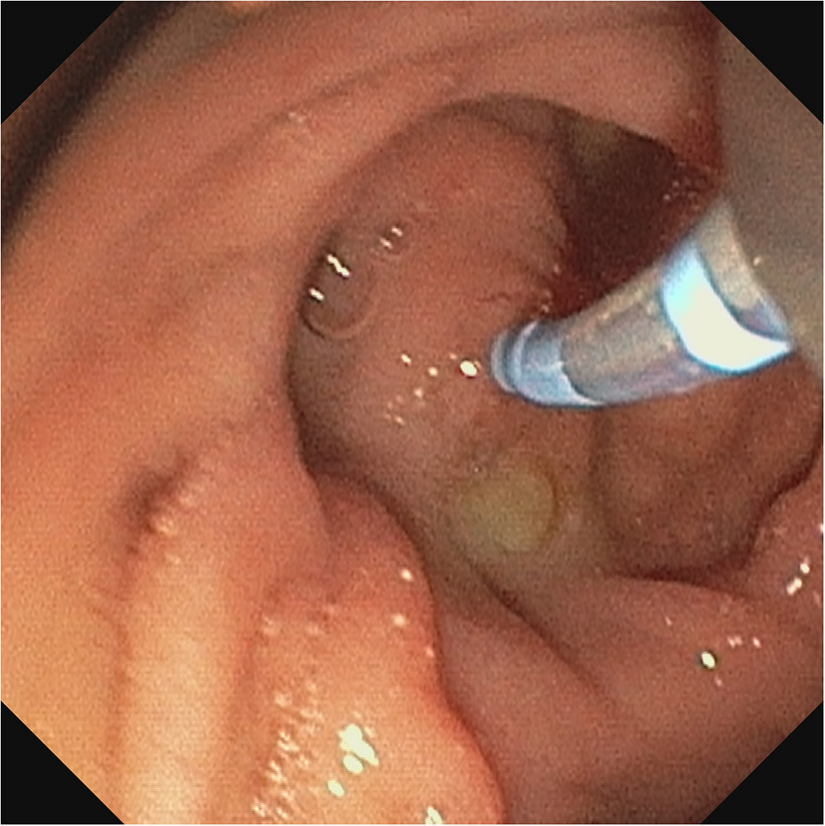
Papillary diverticulum with intubated papilla dorsally rotated and luminal prolapsed.

The following day, the patient presented with significantly elevated total bilirubin levels of 16.38 milligrams per decilitre due to an insufficiently secreting T-drain. Immediate surgical revision was performed, during which the T-drainage was found to be obstructed and was therefore removed. The site of resection and debridement appeared to be in stable condition, and no infectious progress was noted, so no further debridement was required. A larger T-drain was inserted, and bilirubin levels decreased rapidly over the next couple of hours. An additional ERCP revealed stenosis of the common bile duct due to a parapapillary diverticulum (Figure 3). Therefore, papillotomy and stent placement was performed.

Microbiological examination revealed the presence of *Clostridium perfringens* and *Enterococcus faecium* in both blood culture and intraoperative swab.

Postoperatively, as the initial calculated anti-infective therapy, a combination of piperacillin/tazobactam (4.5 g, three times a day for 17 days), vancomycin (sensitive for *E. faecium*, 2 g continuously over 24 h for 12 days) and Amphotericin B (375 mg, once a day for 5 days) was administered. Following the identification of *Clostridium perfringens* that were susceptible to clindamycin, the antibiotic regimen was updated to include clindamycin (900 mg, three times a day). After further testing indicated that *Clostridium perfringens* were also responsive to piperacillin/tazobactam, clindamycin was discontinued after being administered for 6 days. In the further course, vancomycin was replaced by linezolid (600 mg, twice a day for 8 days).

In the further clinical course, despite the T-drain functioning correctly, an increase of CRP and cholestasis enzymes (ALP and gamma-GT) raised concerns for cholangitis. To address this, we intensified the anti-infective therapy by replacing piperacillin/tazobactam with meropenem (1 g, three times a day for 9 days) and caspofungin (70 mg once on the first day, followed by 50 mg once a day for the subsequent 2 days) while also blocking the T-drain. Following a reduction in cholestatic enzymes and infection values, the T-drain was extracted, while the biliary stent was left in position.

Moreover, aside from the antibiotic therapy, differentiated volume and vasopressor therapy in the setting of sepsis was a major challenge, especially due to severe chronic heart failure, developed from 3-vessel coronary heart disease and a history of cardiac infarction.

**Figure 4:**
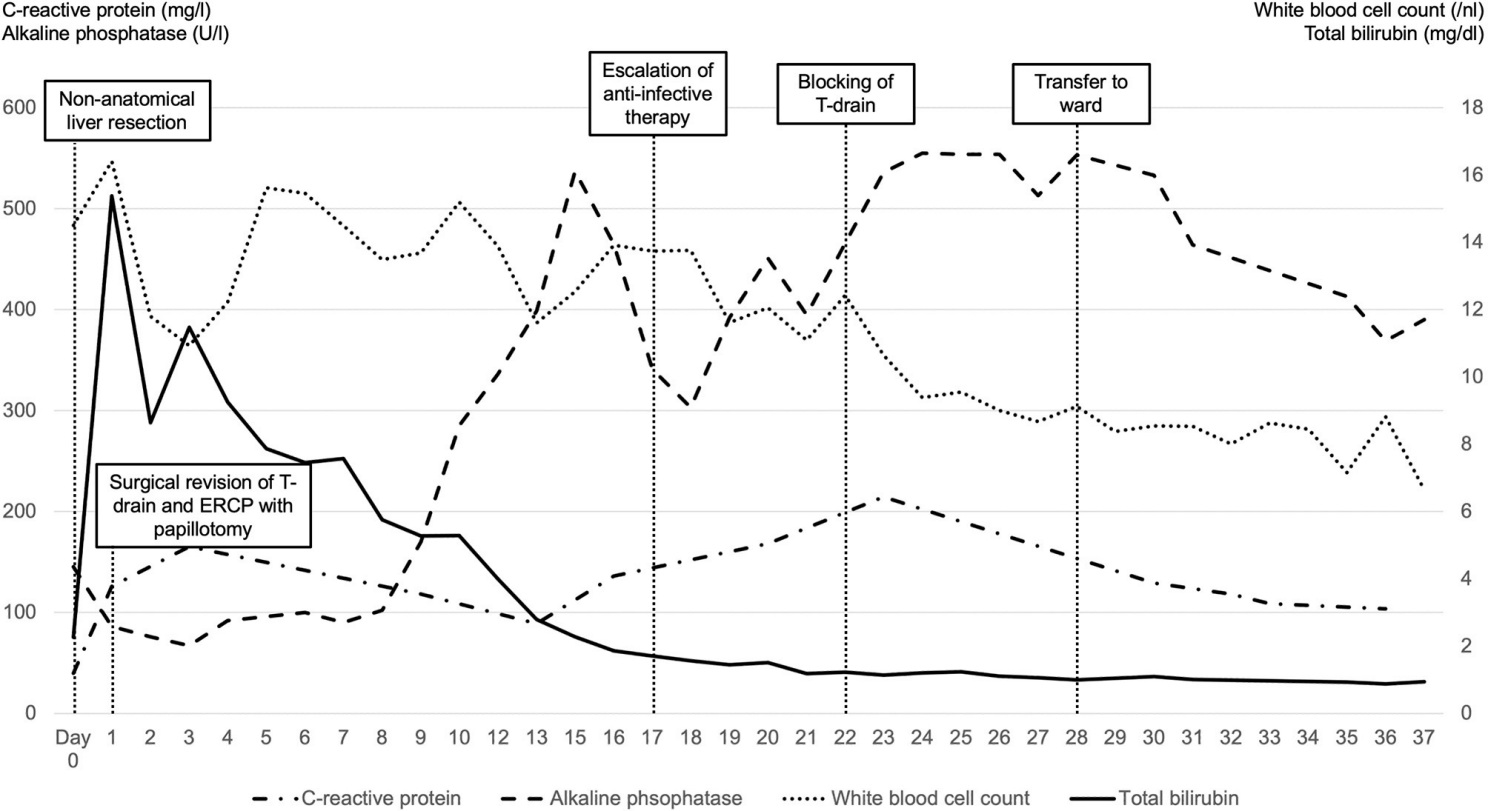
Clinical course with relevant laboratory tests and crucial time points.

After 28 days of intensive care therapy, the patient could be transferred to the surgical ward. However, due to respiratory insufficiency caused by pleural effusion, the patient was readmitted to the intensive care unit for 2 days, where he was stabilized. He did not present any further increase in the white blood cell count, liver parameters, or renal retentions markers. Therefore, the patient could be further mobilized and was transferred to a geriatric rehabilitation site 37 days after admission, fully mobilized and in very good general condition. Figure 4 provides an overview of the clinical course with relevant laboratory tests.

## Discussion

Blachar et al. reported the first case of emphysematous hepatitis in 2002 with a fatal outcome 3 days after admission (1). Other reports followed (2–10), with a maximum survival of 8 days after hospitalization (9). Ghosn et al. reported the successful treatment of a 38-year-old patient with radiological features of emphysematous hepatitis (11). Yet, surgical exploration showed the presence of pus, better fitting the diagnosis of a liver abscess rather than emphysematous hepatitis (11). Three other reports of successfully treated patients with emphysematous hepatitis followed by Estébanez-Ferrero et al. (12), Francois et al. (13), and Pan et al. (14), as summarized in Table 1.

**Table 1:** Case reports of successfully treated emphysematous hepatitis.

**References**	**Year**	**Age**	**Sex**	**Medical history**	**Treatment**	**Pathogens**
Ghosn et al. (11)	2019	38	F	Diabetes mellitus, cholecystectomy, two C-sections, mesh-repair of ventral abdominal hernia	Laparotomy with debridement; antibiotics (amoxicillin, clavulanic acid and tigecycline)	*Escherichia coli* and *Enterococcus faecium*
Estébanez-Ferrero et al. (12)	2021	67	F	No relevant medical history	Laparotomy with debridement and placement of catheter; antibiotics	*Escherichia coli*
Francois et al. (13)	2022	70	F	Diabetes mellitus, cholecystectomy, heterozygote alpha-1-antitrypsin deficiency	Antibiotics (meropenem, vancomycin and amikacin, then rationalized to ceftriaxone and metronidazole); drainage; ERCP with sphincterotomy of the papilla of Vater	*Escherichia coli, Streptococcus anginosus*, and *Klebsiella oxytoca*
Pan et al. (14)	2023	48	M	Diabetes mellitus, hypertension	Antibiotics (ceftriaxone, then rationalized to cefoperazone and sulbactam); drainage	*Klebsiella oxytoca*
This case	2023	79	M	Diabetes mellitus, hypertension, hyperlipidemia, atrial fibrillation, chronic heart failure	Laparotomy with cholecystectomy, debridement and atypical liver resection, bile duct revision with insertion of a T-drain; surgical revision of T-drain; ERCP with papillotomy and stenting; Antibiotics (piperacillin/tazobactam, vancomycin, Amphotericin B, clindamycin, linezolid, meropenem, and caspofungin)	*Clostridium perfringens* and *Enterococcus faecium*

Most reported cases of emphysematous hepatitis, including our patient, show an association with diabetes (1, 3, 7, 9, 11, 14), which also appears to be a risk factor for other abdominal emphysematous diseases such as emphysematous cholecystitis or pyelonephritis (15). Recent abdominal interventions or surgeries also appear to be risk factors (1, 2, 4, 6, 10), which were not known for our patient. It is noteworthy that three cases had a history of hilar bile duct carcinoma, which was treated either by bile duct intervention, radiotherapy, or surgery—with the clinical picture of emphysematous hepatitis, all associated with *Clostridium perfringens*.

Reported pathogens associated with emphysematous hepatitis are *Klebsiella pneumoniae* (1, 5, 9, 10)*, Enterobacter cloacae* (2)*, Streptococcus mutans* (6)*, Enterococcus faecalis* (6, 10)*, Enterococcus faecium* (11)*, Aeromonas ichtiosmia* (10)*, Clostridium perfringens* (2, 4, 7, 10)*, Escherichia coli* (4, 8, 10–13)*, Streptococcus anginosus* (13) and *Klebsiella oxytoca* (13, 14). Successfully treated cases revealed *Escherichia coli* (11–13), *Enterococcus faecium* (11), *Streptococcus anginosus* (13), and *Klebsiella oxytoca* (13, 14) as associated pathogens. In our case, *Clostridium perfringens* and *Enterococcus faecium* were detected in the intraoperative swab as well as in the blood culture.

*Clostridium perfringens* is a Gram-positive, anaerobic bacterium with the ability to produce more than 20 toxins and a remarkably short generation time of 12–17 min at 37°C (16–18). Associated bacteremia shows mortality rates from 26.9% (19) to 74% in septic patients with a rare but well-known complication of massive hemolysis (20). Reported cases of emphysematous hepatitis with *Clostridium perfringens* in blood culture showed a maximal survival time of 3 days (4).

On this basis, the prognosis of our patient seemed to be poor mainly due to the presence of *Clostridium perfringens*. Regarding the bacterial spectrum, all successfully treated cases (11–14), which were nevertheless challenging to treat due to their microbiological diversity, appeared to have a more favorable prognosis due to their bacterial spectrum and the lower age of the patients. Not to mention that one case (11) resembled a liver abscess rather than emphysematous hepatitis.

The eradication of the gas-forming *Clostridium perfringens* requires early surgical resection in combination with prolonged administration of antibiotics, whereas a non-surgical approach, as performed by Francois et al. (13) and Pan et al. (14), would very likely have resulted in a rapid fatal outcome in our patient, especially considering the presence of septic, toxin-induced shock.

As in our case, the initial clinical presentation of emphysematous hepatitis is often inconspicuous and progresses rapidly, as demonstrated clinically and radiologically by the rapid volume expansion of focal gas collections. Considering the size of the affected liver parenchyma, we successfully intervened at a very early stage. Therefore, early abdominal imaging, preferably contrast-enhanced CT with its high sensitivity and specificity in the detection of abnormal gas (15), is crucial for the outcome of patients with emphysematous hepatitis. As therapy, we consider that a “hit hard and early” approach is essential.

## Conclusion

Emphysematous hepatitis remains a challenging disease to diagnose and treat. Despite early and radical surgical therapy with early systemic antibiosis, the disease is often lethal.

## Data availability statement

The original contributions presented in the study are included in the article/supplementary material, further inquiries can be directed to the corresponding author.

## Ethics statement

Written informed consent was obtained from the patient's wife for the publication of this case report.

## Author contributions

CB wrote the original draft and performed the diagnosis in the CT scan. A-KB wrote the original draft and assisted with the first surgery. CS was responsible for conceptualization, edited the manuscript, and indicated and performed the surgery. SA was a supervisor responsible for the ICU patient and edited and reviewed the manuscript. DK, BH, and KB edited and reviewed the manuscript. All authors contributed to the article and approved the submitted version.
